# Coexistence of topological Dirac fermions on the surface and three-dimensional Dirac cone state in the bulk of ZrTe_5_ single crystal

**DOI:** 10.1038/srep40327

**Published:** 2017-01-09

**Authors:** Arnab Pariari, Prabhat Mandal

**Affiliations:** 1 Saha Institute of Nuclear Physics, HBNI, 1/AF Bidhannagar, Calcutta 700 064, India

## Abstract

Although, the long-standing debate on the resistivity anomaly in ZrTe_5_ somewhat comes to an end, the exact topological nature of the electronic band structure remains elusive till today. Theoretical calculations predicted that bulk ZrTe_5_ to be either a weak or a strong three-dimensional (3D) topological insulator. However, the angle resolved photoemission spectroscopy and transport measurements clearly demonstrate 3D Dirac cone state with a small mass gap between the valence band and conduction band in the bulk. From the magnetization and magneto-transport measurements on ZrTe_5_ single crystal, we have detected both the signature of helical spin texture from topological surface state and chiral anomaly associated with the 3D Dirac cone state in the bulk. This implies that ZrTe_5_ hosts a novel electronic phase of material, having massless Dirac fermionic excitation in its bulk gap state, unlike earlier reported 3D topological insulators. Apart from the band topology, it is also apparent from the resistivity and Hall measurements that the anomalous peak in the resistivity can be shifted to a much lower temperature (*T* < 2 K) by controlling impurity and defects.

The low-dimensional pentatellurides, ZrTe_5_ and Hf Te_5_, synthesized in 1973^1^, exhibit a peak in the resistivity (*ρ*) as a function of temperature^2^. This anomaly in the resistivity has been observed at T_*P*_ ~ 145 K for ZrTe_5_ and T_*P*_ ~ 80 K for Hf Te_5_, however, the exact temperature varies from sample to sample depending on the impurity level^3^. With decreasing impurity, the anomalous peak in resistivity shifts to lower temperature^3^. Recent works on ZrTe_5_ reported T_*P*_ as low as ~60 K, which has been ascribed to very low defect and impurity concentration in the samples^4^,^5^. Most of the earlier works have been directed towards understanding the origin of this peak. The charge carrier switches from holes at *T* > T_*P*_ to electrons for *T* < T_*P*_, which is reflected in the sign change of thermoelectric power^6^ and Hall coefficient^7^. Initially, it was believed that this resistive anomaly arises due to a charge-density wave transition, similar to that occurs in NbSe_3_^8^. But the absence of lattice modulation, etc., eliminate the idea of charge density wave formation in ZrTe_5_^9^. Subsequently, the concept of polaronic conduction^10^, semimetal-semiconductor phase transition^11^ and so on, have emerged until a recent theoretical work suggests that the monolayer of ZrTe_5_ and Hf Te_5_ is the most promising candidate for the quantum spin Hall due to the large bulk gap^12^. Suddenly, a material known for its large thermoelectric power^6^, resistivity anomaly^1^ and large positive magnetoresistance^13^, has been brought to our attention to study the topological properties of relativistic Dirac fermion in condensed matter physics^4^,^5^,^14^,^15^,^16^,^17^,^18^,^19^,^20^,^21^.

It has been established from the recent angle resolved photoelectron spectroscopy (ARPES) measurement that the temperature dependence of the electronic band structure across the Fermi energy is responsible for the anomalous peak in resistivity^14^. However, ZrTe_5_ is not free from debate, facing a bigger question. Theoretical calculation shows that electronic band topology of bulk ZrTe_5_ is very sensitive to the lattice parameters. Depending on the values of lattice parameters it can be either a weak or a strong three-dimensional topological insulator^12^. On the other hand, ARPES^4^,^14^, infrared spectroscopy^5^,^15^ and magneto transport^4^ studies show three-dimensional linear dispersion with a small semiconducting gap between the valence and conduction band, i.e., 3D Dirac fermionic excitation with a small mass gap. Do theory and experiment contradict each other or the topological Dirac fermions on the surface and three-dimensional Dirac cone state in the bulk can coexist simultaneously in ZrTe_5_? If the later is possible, it would be a remarkable phenomenon. We will have a three-dimensional topological insulator with Dirac fermionic excitation in its bulk.

## Results

### Crystal structure

**Figure 1:**
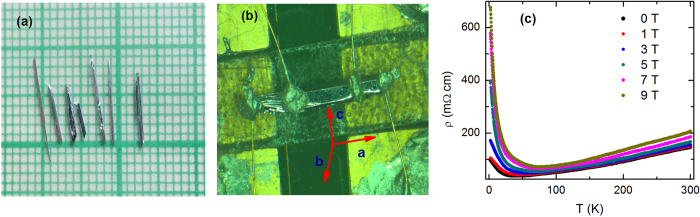
(**a**) Typical size and morphology of few representative single crystals of ZrTe_5_, (**b**) Different crystallographic directions, and (**c**) Temperature dependence of resistivity (*ρ*) both in presence and absence of external magnetic field.

High quality single crystals of ZrTe_5_ were grown by iodine vapor transport method similar to that reported earlier^22^. Typical size and morphology of few representative single crystals are shown in Fig. 1(a). Phase purity and the structural analysis of the samples were done by high resolution powder x-ray diffraction (XRD) technique (Rigaku, TTRAX II) using Cu-K_*α*_ radiation [see Supplementary Figure S1]. Within the resolution of XRD, we have not seen any peak due to the impurity phase. The calculated value of the lattice parameters are *a* = 3.96 Å, *b* = 14.50 Å and *c* = 13.78 Å with space group symmetry *Cmcm*, similar to the earlier reports^23^,^24^,^25^. The structure of the pentatellurides consists of trigonal prismatic chains of ZrTe_3_ along **a** axis connected via parallel zigzag chains of Te atoms along the **c** axis, which together form 2D planes weakly bonded via van der Waals force along the **b** axis^12^. Figure 1(b) shows the crystallographic directions of a typical ZrTe_5_ single crystal.

### Temperature dependence of resistivity both in presence and absence of external magnetic field

Resistivity and transverse magnetoresistance measurements are done by applying current along the **a** axis and magnetic field perpendicular to the **ac** plane, i.e., along **b** axis. Figure 1(c) shows the temperature dependence of resistivity of ZrTe_5_ single crystal both in presence and absence of magnetic field. The zero-field *ρ* exhibits metallic behavior (d*ρ*/d*T* > 0) down to 25 K. Below 25 K, *ρ* shows a weak upturn, i.e., a crossover from metallic to semiconducting like behavior. However, several earlier reports show that a broad peak appears in the temperature dependence of *ρ*, which is known as the resistivity anomaly of ZrTe_5_^1^,^2^,^3^,^4^,^5^. We have already mentioned that the temperature at which *ρ* shows peak varies widely; from 60 K to 170 K depending on the presence of impurity and defect concentration in the sample. It has been argued that the binding energy shift of the valence and conduction bands as a function of temperature is responsible for the peak at *T*_*P*_^14^. The sign of the charge carrier changes from positive (hole) to negative (electron) and the peak in *ρ*(*T*) appears when the chemical potential crosses the gap (~50 ± 10 meV) from valence band to conduction band. The absence of resistivity peak down to 2 K in the present sample could be attributed to much smaller impurities and defects. Under application of magnetic field, *ρ* increases sharply at low temperature and the metal-semiconductor crossover shifts to higher temperature, which are consistent with the earlier reports^4^,^25^. But, no re-entrant metallic state has been observed up to 9 T.

### Hall resistivity and transverse magnetoresistance

**Figure 2:**
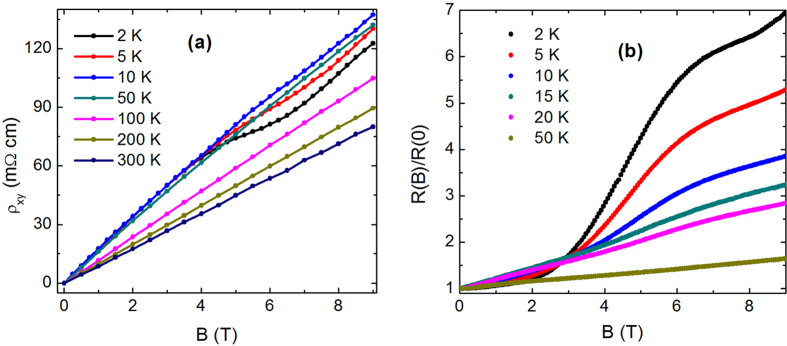
(**a**) Hall resistivity (*ρ*_*xy*_) of ZrTe_5_ single crystal at several representative temperatures over the range 2–300 K. (**b**) Transverse magnetoresistance (*I*⊥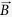) normalized to the zero field value upto 9 T.

To further ensure the absence of resistivity anomaly, which has been ascribed to the switching of *p*-type semimetal to *n*-type semimetal state, we have done Hall measurements down to 2 K. Figure 2(a) shows that the Hall resistivity (*ρ*_*xy*_) remains positive over the entire temperature range 2–300 K. The absence of sign change in *ρ*_*xy*_ is consistent with the observed *T* dependence of *ρ*. One can see that *ρ*_*xy*_ is linear over the entire field range except at low temperature, where an upward curvature appears at high fields due to the Shubnikov-de Haas oscillations. A systematic increase of the slope of the Hall resistivity with decreasing temperature is consistent with the temperature evolution of electronic band structure in ZrTe_5_^14^,^21^. From the slope of *ρ*_*xy*_(*H*), the bulk carrier density (*n*) is calculated to be ~4 × 10^16^ cm^−3^ and ~7 × 10^16^ cm^−3^ at 2 and 300 K, respectively. We would like to mention that the carrier density in the present crystal is almost one order of magnitude smaller than the earlier reported ones^18^,^26^. Figure 2(b) shows the normalized magnetoresistance (MR) up to 9 T magnetic field. MR is large, positive and shows no sign of saturation in the measured temperature and field range. The observed behavior of MR is similar to the earlier reports^13^,^16^,^17^. At low temperature, MR is dominated by a very low frequency (~3 T) Shubnikov-de Haas oscillation, which implies the presence of a tiny Fermi pocket, consistent with the low carrier density determined from the Hall measurements. Employing the Onsager relation *F* = (*ϕ*_0_/2*π*^2^)*A*_*F*_, we have calculated the cross-sectional area (*A*_*F*_) of the Fermi surface normal to the field ~6.2 × 10^−5^ *Å*^−2^. At high temperature, where the quantum oscillation suppresses, MR becomes linear.

### Longitudinal magnetoresistance and Chiral anomaly

As proposed by Hermann Weyl in 1929, the four-component massless Dirac equation in three spatial dimensions can be separated into two two-component equations, 
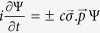
, where 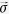 and 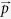 are the Pauli matrices and momentum respectively. The above equation describes particles with a definite chirality 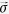.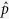, known as Weyl fermions. according to the classical equation of motion the number of fermions with plus or minus chirality is conserved separately. But, the relativistic theory of charged chiral fermions in three spatial dimensions holds the so-called chiral anomaly- non-conservation of chiral charge induced by external gauge fields with non-trivial topology, known as Adler-Bell-Jackiw anomaly^27^,^28^. Nielsen and Ninomiya provided a physical picture of the chiral anomaly in the context of condensed matter physics^29^. Considering a band structure in three-dimension which supports two Weyl nodes with opposite chirality separated in momentum space and applying a magnetic field along the line joining the Weyl nodes, they predicted an enhanced magneto-conductance due to the charge pumping from one node to another in presence of an electric field (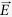) parallel to 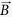.

In 3D Dirac semimetals, a four-component massless Dirac fermion is nothing but the two copies of distinct Weyl fermions. The application of magnetic field splits the four-fold degenerate Dirac node into two Weyl nodes of opposite chirality, along the direction of magnetic field^30^,^31^. Initially, the plus and minus chirality fermions in the different Weyl nodes have same chemical potential *μ*^+^ = *μ*^−^. Whereas, 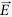 parallel to 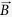 creates an imbalance (*μ*^+^ ≠ *μ*^−^) between the two Weyl nodes with opposite chirality, which induces a charge pumping from one Weyl node to another. The net current generation due to the chiral imbalance is j_*c*_ = 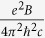
(*μ*^+^ − *μ*^*−*^)^4^,^31^. Again, (*μ*^+^ − 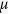
^−^) is proportional to 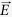.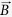. As a result, the enhanced magneto-conductance is expected to show quadratic *B* dependence in the form, *σ*_*c*_ = *σ*_0_ + *a*(*T*).*B*^2^, where *σ*_0_ is the zero-field conductivity. The field independent constant, *a*(*T*) has the inverse *T*^ 2^ dependence,


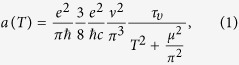


where *ν, τ*_*v*_ and *μ* are the Fermi velocity, chirality changing scattering time and chemical potential, respectively^4^. But, in addition to the negative quadratic MR associated to chiral anomaly, small positive MR components associated to different physical phenomenon may contribute to transport^32^,^33^,^34^. Conventional nonlinear band contribution around the Fermi level, which has the field dependence, 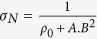, is common^32^,^33^,^34^. As a result, the longitudinal magneto-conductance can be fitted with the theoretical expression, 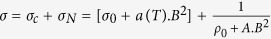.

**Figure 3:**
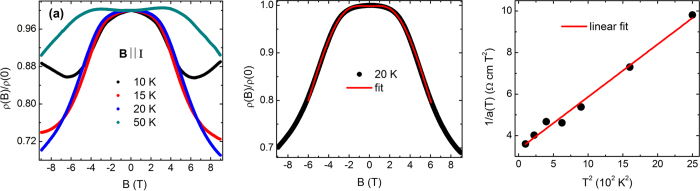
(**a**) Magnetoresistance measured at temperatures from 10 to 50 K, when applied current and magnetic field are parallel to each other, (**b**) Magnetoresistance at 20 K, fitted with the theoretical expression, 
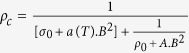
, and (**c**) Temperature dependence of 1/*a*, where *a* is in the units of S cm^−1^ T^−2^.

To probe the chiral anomaly, we have measured longitudinal magnetoresistance (LMR) by applying both the current and magnetic field along the **a** axis. As shown in Fig. 3(a), the resistance at 10 K gradually decreases with increasing field until an upturn occurs at high field. A small positive transverse MR component due to unavoidable misalignment in parallel configuration is responsible for this high field upturn. As the positive MR component rapidly suppresses with increasing temperature, the longitudinal negative MR becomes more clearly visible at higher temperatures. LMR at several other temperatures from 5 to 40 K, has been shown in Supplementary Figure S2. With further increase in temperature above 40 K, however, the negative MR itself becomes very weak. The negative LMR was reproduced by several independent measurements and also verified in different crystals. Chiral anomaly induced negative LMR has also been observed in earlier magneto-transport studies in ZrTe_5_^4^,^34^. The nature and strength of negative LMR in the present sample are comparable to that reported earlier^4^,^34^. LMR has been fitted with the inverse of the above mentioned theoretical expression for the longitudinal magneto-conductance and shown at a temperature 20 K in Fig. 3(b). A good fitting between the theoretical expression and experimental data is reflected in the above mentioned figure. By fitting LMR at different temperatures in the range 10–50 K [see Supplementary Figure S2 for the theoretical fitting at several representative temperatures], we have calculated the values of the parameter *a*. Observed value of *a*(*T*) indicates that the strength of induced chiral current for the present sample is comparable with the earlier report on ZrTe_5_^4^. In Fig. 3(c), we have plotted *a*^−1^ vs *T *^2^. One can see from the figure that *a*^−1^ is almost linear in *T*^2^, as predicted theoretically. Thus, the negative longitudinal MR in 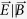 configuration implies four-component massless Dirac fermionic excitation in the bulk state of ZrTe_5_ single crystal^4^,^34^. It may be noted that the negative MR due to induced chiral anomaly is a well established phenomenon in three-dimensional Dirac semimetals, which has also been observed in Cd_3_As_2_^31^ and Na_3_Bi^35^. Presence of a small gap between the upper and lower Dirac cone in bulk may reduce the magnitude of the chiral current, but cannot destroy it fully^4^.

### Signature of topological surface state from magnetization measurements

The low-energy physics of the surface state for a three-dimensional topological insulator can be described by the Dirac type effective Hamiltonian, H_*sur*_(*k*_*x*_, *k*_*y*_) = *ħv*_*F*_(*σ*^*x*^*k*_*y*_ − *σ*^*y*^*k*_*x*_), where *v*_*F*_ is the Fermi velocity^36^,^37^. Thus, for a fixed translational momentum 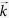, the “spin”, denoted by the Pauli matrix 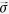, has a fixed direction for the eigenstate of the Hamiltonian. This is the most important property of the nontrivial topological surface state in 3D topological insulators, known as “spin-momentum locking”. As the “spin” is always perpendicular to the momentum, one can introduce a helicity operator for the spin texture on circular constant energy contour of the Dirac cones^37^, 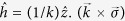. This leads to left-handed spin texture for the upper Dirac cone and right-handed spin texture for the lower Dirac cone in the momentum space. Whereas at the Dirac point, as long as the Dirac spectrum is not gapped, the electron spin should be free to align along the applied magnetic field due to the singularity in spin orientation^38^. This predicts a low-field paramagnetic peak in the susceptibility curve *χ*(*H*).

**Figure 4:**
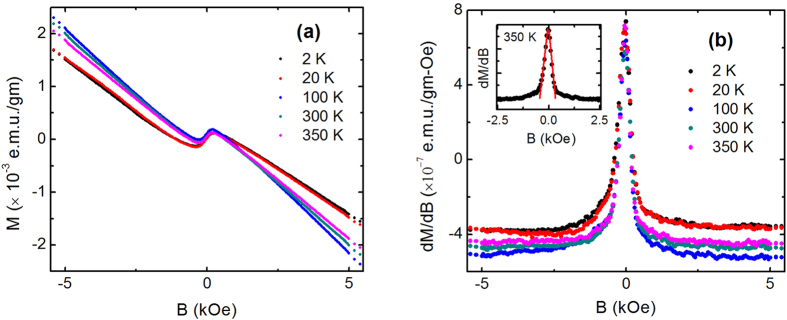
(**a**) Magnetization of ZrTe_5_ single crystal, measured at several representative temperatures from 2 to 350 K. (**b**) Differential susceptibility 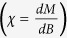 obtained after taking derivative of the magnetization with respect to external magnetic field. Inset shows the linear *B* dependence of *χ*, as *B* tending towards zero on both side of the zero field cusp at representative temperature 350 K.

Figure 4(a) shows the magnetization of single crystal of ZrTe_5_ with magnetic field along the **a** axis. Over the whole range of temperature from 2 to 350 K, ZrTe_5_ shows diamagnetic signal except a paramagnetic upturn in the low-field region. It might be worthy to mention that single crystals of standard diamagnetic bismuth and three-dimensional Dirac semimetal Cd_3_As_2_ do not show this type of behaviour [see Supplementary Figure S3 and Figure S4(b)]. On the other hand, similar paramagnetic response has been observed in single crystals of well established three-dimensional topological insulator Bi_1.5_Sb_0.5_Te_1.7_Se_1.3_ [see Supplementary Figure S5]. Figure 4(b) shows that a cusp-like paramagnetic susceptibility sharply rises above the diamagnetic floor in a narrow field range of ~2 kOe around zero field. The height of the peak from the diamagnetic floor and its sharpness are insensitive to the temperature. This singular response of susceptibility shows no sign of thermal rounding up to as high as 350 K (~32 meV), which is almost one-half of the bulk band gap^4^,^21^. Similar robust and singular paramagnetic response have been reported for the family of three-dimensional topological insulators which is the fingerprint of the helical spin texture of the topological Dirac fermions on the surface^38^,^39^. Setting both the chemical potential *μ* and temperature to zero, one can show that, at low field, this paramagnetic Dirac susceptibility decays linearly from its zero-field value^38^ as, 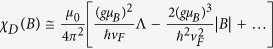. Where *g* is the Landé *g*-factor and Λ is the effective size of the momentum space contributing to the singular part of the free energy. It has been argued^38^ that the maximum of the susceptibility, i.e., the peak height at zero field over the diamagnetic floor, depends on Λ, and thus controlled by the bulk bands. Whereas the nature of the singularity (i.e. cuspiness and linear-in-field decay of susceptibility at low fields, almost absence of thermal smearing, etc.,) is universal to the entire family of 3D topological insulators. Inset of Fig. 4(b) shows the linear fit to the experimental data on the both sides of the zero-field cusp. The linear-in-field decay of the paramagnetic response, even at the highest measuring temperature 350 K, is evident from the figure.

Often, surface states show ageing effect, which has been attributed to surface reconstruction and the formation of two-dimensional electron gas due to the bending of the bulk band at the surface^38^,^39^,^40^,^41^. To see whether such behaviour is visible in the present case, similar measurements have been done on the same pieces of single crystals after three weeks of the first measurement [See Supplementary Figure S6]. Although the nature of the peak and its robustness against temperature remain unaffected, the reduction in peak height over time may be attributed to the expected ageing effect, similar to that observed in Bi_2_Se_3_, Sb_2_Te_3_ and Bi_2_Te_3_^38^. It has been pointed out that the spin/orbit texture may also exist in the bulk state of the material with strong spin-orbit coupling, such as in BiTeI^42^ and WTe_2_^43^. Keeping this information in mind, one may think the possibility of the singular paramagnetic response from the bulk of ZrTe_5_. But, as reported by the earlier ARPES measurements^4^,^14^,^21^, the bulk state of ZrTe_5_ holds a band gap (~50 ± 10 meV) between the upper and lower Dirac cone, which disobeys the primary condition for the singularity in electron spin orientation from the spin/orbit texture. Secondly, the negative longitudinal magnetoresistance due to chiral charge imbalance under non-trivial gauge field and ARPES results, established the presence of four-component massless Dirac fermion in the bulk 3D Dirac cone state of ZrTe_5_. As far as we know, a four-component 3D Dirac fermion originating from the spin-degenerate band, cannot have any spin-orbit texture. The age dependent reduction of the peak height, whereas the diamagnetic back ground is unaffected, also confirms the surface origin of this singular paramagnetic response.

## Discussion

**Figure 5:**
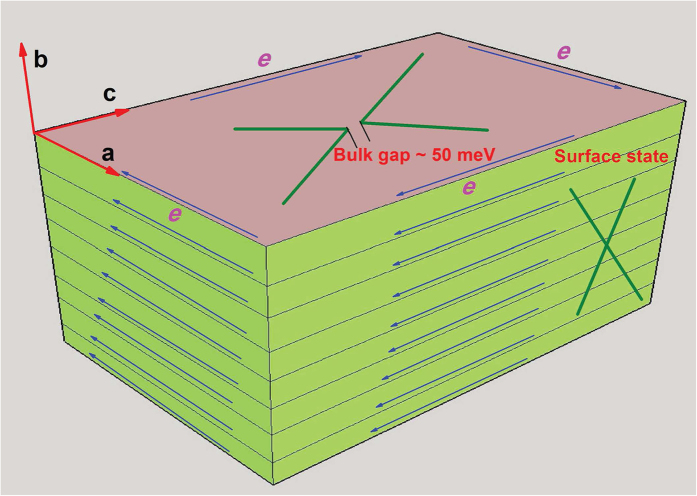
A schematic diagram, representing the minimum nontrivial topological nature of the electronic band structure of ZrTe_5_ single crystal. ZrTe_5_ monolayers, which lie in the **ac**-plane, stack together along the **b**-axis by weak van der Waals attraction. Independent conducting edge state of monolayers, as shown by the blue arrows in the figure, forms a topological surface state in bulk sample, known as weak three-dimensional topological insulating state.

We have detected a robust zero-field paramagnetic peak in the susceptibility of ZrTe_5_ due to the helical spin texture associated with the Dirac fermions of the surface state of the three-dimensional topological insulator. Also, the negative longitudinal magnetoresistance implies induced chiral anomaly in ZrTe_5_, which is the signature of the three-dimensional Dirac fermion in the bulk. This allows one to conclude that ZrTe_5_ is a novel quantum phase of matter, which hosts both topological Dirac fermions on the surface and three-dimensional Dirac cone state with a mass gap between valence and conduction bands in the bulk. As mentioned earlier, ZrTe_5_ can be either a weak or a strong three-dimensional topological insulator depending on the values of the lattice parameters^12^. The simplest kind of topological insulator in three-dimension can be understood by stacking the layers of the 2D quantum spin Hall insulator with weak van der Waals bonding between them^44^, similar to the stacking of monolayers of ZrTe_5_ along **b** axis. A schematic diagram has been shown in Fig. 5, representing the possible minimum nontrivial electronic state in ZrTe_5_ single crystal. Blue arrows represent the conducting edge state of a monolayer, which together form a topological surface state in the bulk sample. On the other hand, the bulk band with semiconducting gap is linear enough to show the signature of massless Dirac fermionic excitation in electronic transport.

In condensed matter electronic system, the topological classes are defined on the basis of the elementary concept, which states that the Hamiltonian cannot be smoothly deformed from one class of materials to another without closing the gap in electronic band structure. As a result, at the interface of the two materials with different topological band gap, there should be a conducting surface state, defined by the TKNN number^44^,^45^. Unless there is a bulk gap, there should be no well-defined topological surface state. So, either band crossing in the bulk, i.e., 3D Dirac node or the 2D Dirac cone surface state can survive. In all 3D topological insulators reported so far, the semiconducting bulk band is highly non-linear and the gap is significantly large (~300 meV) compared to ZrTe_5_^38^,^46^,^47^. That is why the coexistence of 2D Dirac cone surface state and 3D Dirac fermions in the bulk is difficult. To the best of our knowledge, ZrTe_5_ is the only 3D topological insulator in the history of material science, which has Dirac fermionic excitation in the bulk.

### Note added

After completion of the present work [arXiv:1603.05175]. we come across the results of ref. 19. From the scanning tunneling microscopy and angle-resolved photoemission spectroscopy, it has been shown that the monolayer of ZrTe_5_ is a large gap 2D topological insulator, as the theory proposed^12^. The authors have found conducting state at monolayer step edge of **ac**-plane and far away from the edge, i.e., well inside the plane, the spectrum is fully gapped. This is consistent with our proposed nontrivial electronic state for the bulk ZrTe_5_, as shown in schematic Fig. 5. Very recently our results have been confirmed by Manzoni *et al*. through ARPES^48^.

## Methods

A stoichiometric mixture of Zr (Alfa Aesar 99.9%) and Te (Alfa Aesar 99.99%) was sealed in a 15 cm long quartz tube with iodine (~5 mg/cc) and placed in a box furnace. It was then heated for seven days at 520 °C and cooled to room temperature at 10 °C/h. Next, the tube was shifted to a two-zone gradient furnace. One end of the tube containing the product was placed at 540 °C while the other end of the tube was placed at the cooler end of the furnace at 450 °C to provide a temperature gradient for four days. After slowly cooling (~30 °C/h) it to room temperature, single crystals with needlelike morphology were obtained at the cooler end.

The resistivity measurements were done by standard four-probe technique. Electrical contacts were made using conductive silver paste. The electrical transport measurements were carried out in 9 T physical property measurement system (Quantum Design) and cryogen free measurement system (Cryogenic). Magnetization was measured using a Superconducting Quantum Interference Device–Vibrating Sample Magnetometer (SQUID-VSM) (Quantum Design). Before the magnetization measurements, the system was standardized using single crystal of diamagnetic bismuth (Alfa Aesar 99.99%) and paramagnetic palladium [see Supplementary Figure 3 and Supplementary Figure 4(a)].

## Additional Information

**How to cite this article:** Pariari, A. and Mandal, P. Coexistence of topological Dirac fermions on the surface and three-dimensional Dirac cone state in the bulk of ZrTe_5_ single crystal. *Sci. Rep.* **7**, 40327; doi: 10.1038/srep40327 (2017).

**Publisher's note:** Springer Nature remains neutral with regard to jurisdictional claims in published maps and institutional affiliations.

## Supplementary Material

Supplementary Information
