# Supplementary material for: Coexistence of topological Dirac fermions on the surface and three-dimensional Dirac cone state in the bulk of ZrTe5 single crystal
Source: Sci Rep. 2017 Jan 9;7:40327. doi: 10.1038/srep40327 (PMC5220326; doi:10.1038/srep40327)
Supplement: Supplementary Information [file srep40327-s1.pdf]

Supplementary information for "Coexistence of topological Dirac fermions on the surface and three-dimensional Dirac cone state in the bulk of $ZrTe_5$ single crystal"

Arnab Pariari & Prabhat Mandal
Saha Institute of Nuclear Physics, 1/AF Bidhannagar, Calcutta 700 064, India

A. Sample Characterization

Phase purity and the structural analysis of the samples were done with the high-resolution powder x-ray diffraction (XRD) technique (Rigaku, TTRAX II) with Cu-K_α radiation. FIG.S1 shows the x-ray diffraction pattern of powdered sample of $ZrTe_5$ single crystals at room temperature. Within the resolution of XRD, we have not seen any peak due to the impurity phase. Using the Rietveld profile refinement program of diffraction patterns, we have calculated the lattice parameters a=3.96 Å, b=14.50 Å and c=13.78 Å with space group symmetry $Cmcm$.

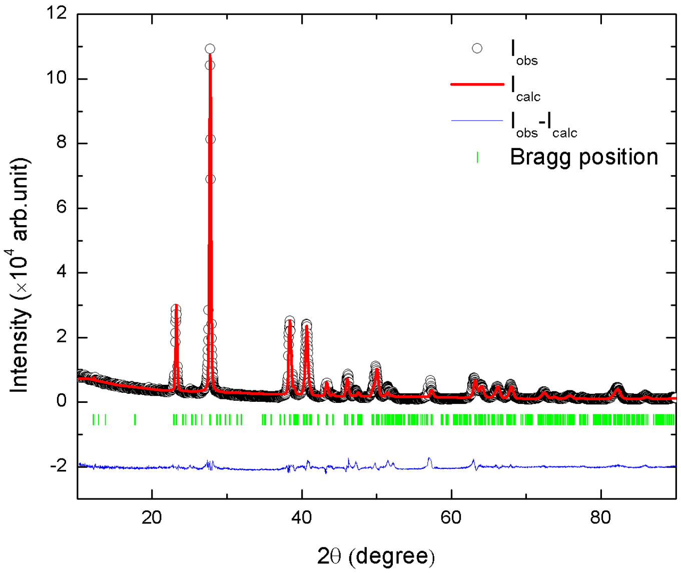

FIG.S 1. (Color online) X-ray diffraction pattern of powdered single crystals of $ZrTe_5$. Black open circle, experimental data; red, the calculated pattern; blue, the difference between experimental and calculated intensities; green, the Bragg positions.

B. Longitudinal magnetoresistance at several representative temperatures

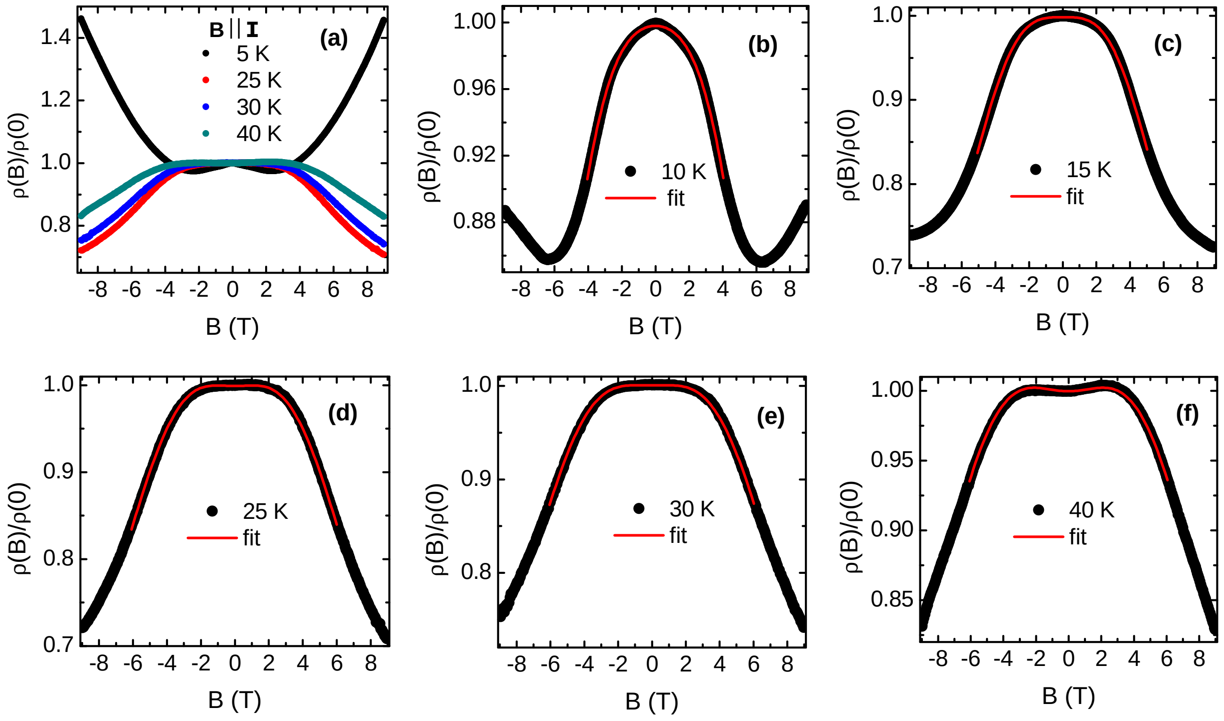

FIG.S 2. (Color online) (a)Magnetoresistance measured at temperatures 5 K, 25 K, 30 K and 40 K, when applied current and magnetic field are parallel to each other. (b), (c), (d), (e) and (f) are the fit to the experimental data with the theoretical expression, $\rho_c=\frac{1}{[\sigma_0+a(T).B^2]+\frac{1}{\rho_0+A.B^2}}$, at several representative temperatures.

C. Magnetic measurements of standard samples

We have done magnetization measurements on standard bismuth and palladium samples in SQUID-VSM [MPMS 3, Quantum Design] prior to $ZrTe_5$ single crystal. FIG.S3 (a) shows that linear diamagnetic moment of bismuth at 2, 10 and 100 K passes through the origin. This is more clear from FIG.S3 (b), which shows magnetic field dependence of differential susceptibility ($\chi=\frac{dM}{dB}$). With quantum oscillation at high fields, absence of any paramagnetic peak around zero field implies that the singular paramagnetic susceptibility in $ZrTe_5$ single crystal is not due to any spurious response in our system.

FIG. S4(a) shows the expected magnetic behaviour of paramagnetic palladium sample, provided by the Quantum

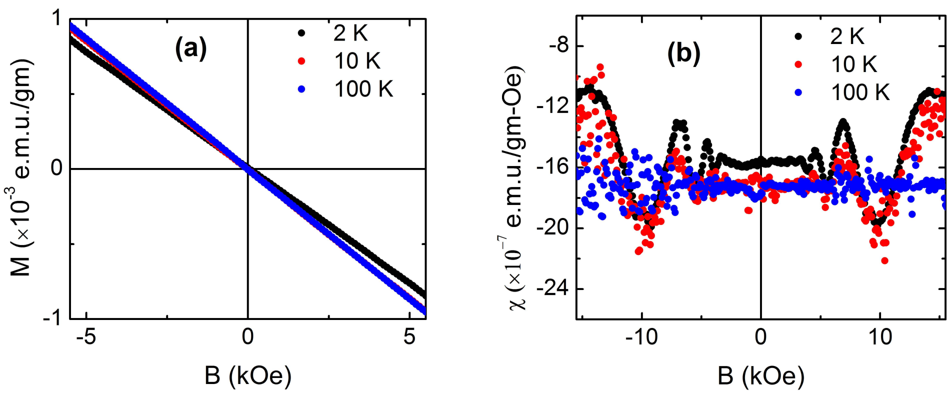

FIG.S 3. (Color online) (a) Magnetization measured in SQUID-VSM (Quantum Design) at several representative temperatures for standard diamagnetic bismuth sample. (b) Differential susceptibility ($\chi=\frac{dM}{dB}$) obtained after taking numerical derivative of the magnetization with respect to external magnetic field.

Design. FIG.S4(b) shows the low-field susceptibility of palladium at 2 K and room temperature. The nonlinear

behaviour of χ at low field and a broad zero field peak at 2 K are completely suppressed at room temperature. This is entirely different from the singular, robust and linear low-field paramagnetic response from the topological surface state in $ZrTe_5$. For the sake of fundamental interest, we have also measured the magnetization of a single crystal of three-dimensional Dirac semimetal Cd_3As_2, as shown in FIG.S4(c). This shows perfect diamagnetic behaviour with no paramagnetic sign at low field.

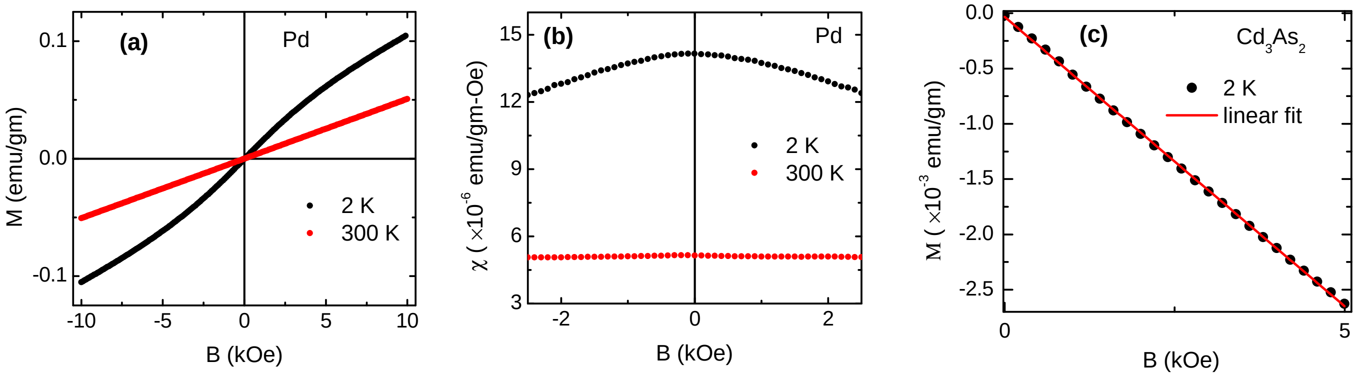

FIG.S 4. (Color online) (a) Magnetization of standard paramagnetic palladium sample at 2 and 300 K, (b) Differential susceptibility ($\chi=\frac{dM}{dB}$) obtained after taking numerical derivative of the magnetization with respect to external magnetic field, and (c) Magnetization of single crystal of three-dimensional Dirac semimetal Cd_3As_2. Solid line implies linear fit to the experimental data.

In this context, we would also like to mention that similar magnetization measurements have been performed on single crystal of well established three-dimensional topological insulator $Bi_{1.5}Sb_{0.5}Te_{1.7}Se_{1.3}$ [1,2] using the same experimental setup. We have observed singular robust paramagnetic susceptibility peak like to that observed in $ZrTe_5$ and shown in FIG.S5(a) and FIG.S5(b) at representative temperatures 5 K and 280 K. The details will be reported as an another independent work.

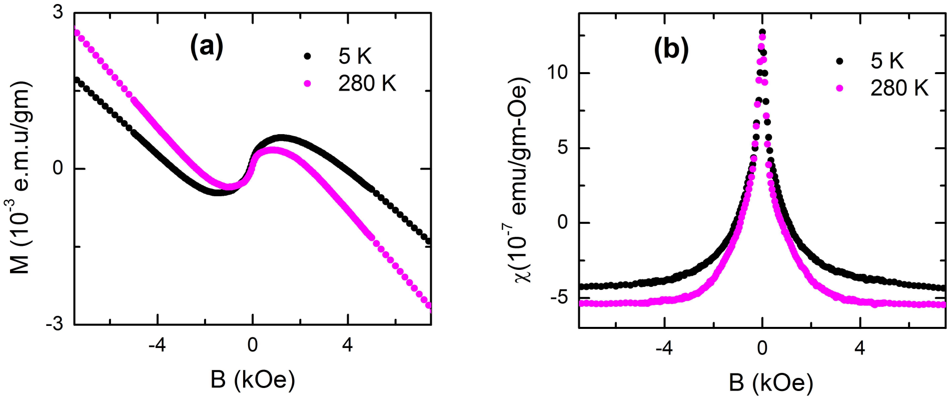

FIG.S 5. (a) Magnetization (M) vs B of freshly cleaved single crystal of $Bi_{1.5}Sb_{0.5}Te_{1.7}Se_{1.3}$ at temperatures 5 K and 280 K, (b) Susceptibility ($\chi=\frac{dM}{dB}$) as a function of B, calculated by taking the first order derivative of magnetization.

D. Aging effect of $ZrTe_5$ single crystal

Figure S6 (a) shows the magnetization of $ZrTe_5$ single crystal after three weeks from the first measurements [Fig. 4(b)]. As shown in Fig. S6 (b), the singular paramagnetic response remains as robust as the earlier measurements [Fig. 4(b) and Fig. S6 (c)] against temperature. It also retains the linear in field decay on the both sides of the zero-field cusp, as shown in the inset of Figure S6 (b) at a representative temperature 350 K. But the overall cusp height has been reduced over time. Similar reduction in peak height has been observed in Bi_2Se_3, Sb_2Te_3 and Bi_2Te_3 [3] and has been attributed to surface reconstruction and the formation of two-dimensional electron gas due to the bending of bulk band at the surface [3-5].

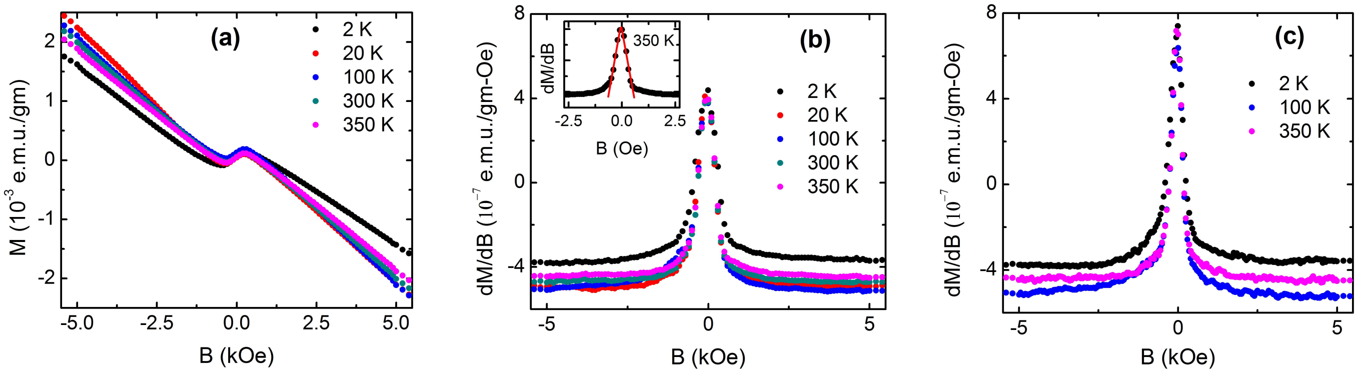

FIG.S 6. (Color online) (a) Magnetization of $ZrTe_5$ single crystal, which was kept for three weeks in desiccator from the first measurements [Fig.4(b)]. (b) Differential susceptibility ($\chi = \frac{dM}{dB}$) obtained after taking derivative of the magnetization with respect to external magnetic field. Inset shows the linear B dependence of χ as B tending towards zero on both sides of the zero field cusp at representative temperature 350 K. (c) Susceptibility of the earlier measurements [Fig.4(b)] is shown to compare the nature of the peak.

References

1. Arakane, T. et al. Tunable Dirac cone in the topological insulator $Bi_{2-x}Sb_xTe_{3-y}Se_y$. Nat. Commun. **3**, 636 (2012).

2. Kim, S. et al. Robust Protection from Backscattering in the Topological Insulator $Bi_{1.5}Sb_{0.5}Te_{1.7}Se_{1.3}$. Phys. Rev. Lett. **112**, 136802 (2014).

3. Zhao, L. et al. Singular robust room-temperature spin response from topological Dirac fermions. Nat. Mater. **13**, 580 (2014).

4.He, X. et al. Surface Termination of Cleaved Bi_2Se_3 Investigated by Low Energy Ion Scattering. Phys.Rev.Lett. **110**, 156101(2013).

5. Bahramy, M. S. et al. Emergent quantum confinement at topological insulator surfaces. Nat. Commun. **3**,1159(2012).
